# Nasopharyngeal Airway in the Trachea: A Case Report

**DOI:** 10.7759/cureus.60149

**Published:** 2024-05-12

**Authors:** Muhammad Usman Khan, Waqar Khalid, Naveed Latif, Tanvir Hussain

**Affiliations:** 1 Anesthesiology and Critical Care, National Hospital & Medical Center, Lahore, PAK

**Keywords:** airway management, foreign body airway obstruction, difficult airway management, acute airway obstruction, airway foreign body, tracheal foreign body, nasopharyngeal airway

## Abstract

We report a case of a cannulated nasopharyngeal airway (NPA) in a patient having a neurological deficit, absent gag reflex, and no clinically obvious signs of respiratory distress. The patient had two episodes of vomiting before admission and was admitted with the initial working diagnosis of aspiration pneumonia; however, a preliminary chest X-ray (CXR) revealed an NPA, sitting vertically in the airway. It is our emphasis that thorough clinical history and radiological imaging are of paramount importance in prompt management of such airway complications.

## Introduction

The inadvertent migration of a nasopharyngeal airway (NPA) into the trachea is an uncommon yet critical complication that requires immediate management as it may result in clinically significant acute respiratory distress, therefore, making a recognizable diagnosis of airway obstruction [1].

Clinical history and examination on presentation are necessary for tracing the clinical course. Additionally, imaging modalities such as chest X-ray (CXR) may facilitate early recognition and localization of airway foreign bodies.

## Case presentation

An 85-year-old male with a previously known case of diabetes, hypertension, and left-sided stroke was received in the Emergency Department after having two episodes of vomiting at home. The patient had GCS of 10/15 (E4V1M5), a heart rate of 115 bpm (regular), NIBP of 160/90 mmHg, and Spo_2_ 96% at room air with no signs of tachypnea, stridor, or respiratory distress. Further examination revealed bilateral rales and ronchi.

Initial management involved nebulization with ipratropium, beclomethasone, and albuterol. An injection of 100 mg of hydrocortisone was also given. All baseline tests (CBC, LFT, RFT, S/E, CRP, and urine RE) along with a CXR were ordered and an initial working diagnosis of aspiration pneumonia was made before the patient was shifted to the ICU.

**Figure 1:**
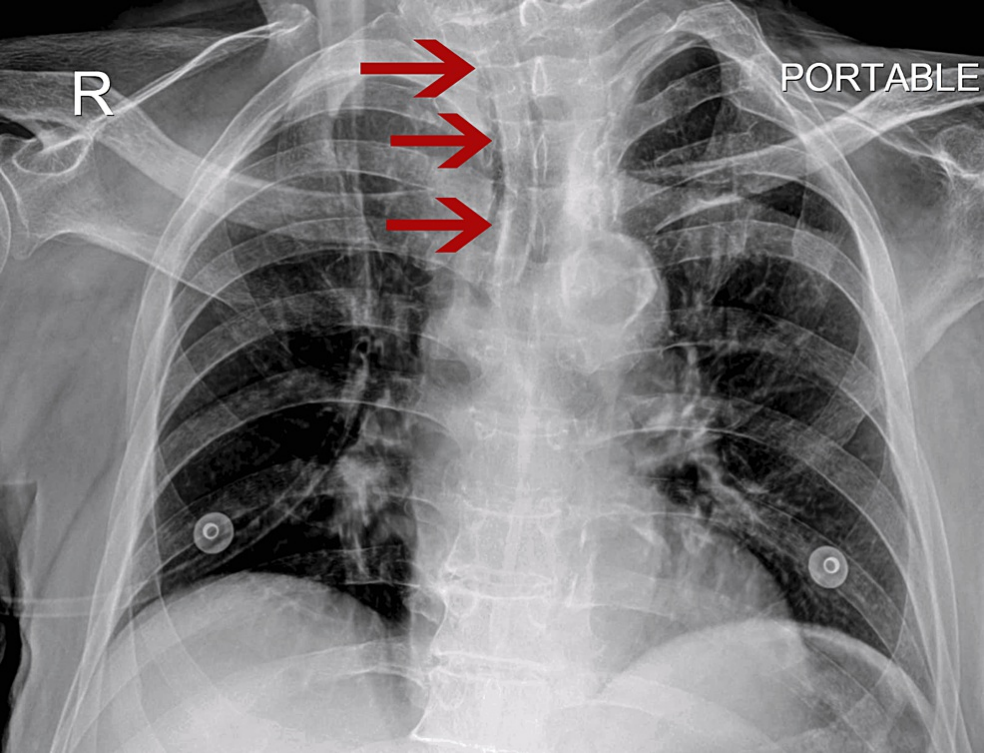
CXR on admission to ICU showing cannulated NPA approximately 2 cm above the carina NPA, nasopharyngeal airway; CXR, chest X-ray

The patient had a history of dysphagia, absent gag reflex, and had a PEG tube placed one and a half years back, which was changed three weeks before the current admission. There was a prolonged history of being immobile and was receiving home nursing care. Despite this, there were several hospital admissions throughout the year due to aspiration pneumonia. Upon arrival in the ICU, the preliminary examination was done and showed GCS 10/15 (E4V1M5), a heart rate of 91 bpm (regular), NIBP of 146/63 mmHg, and Spo_2_ 94% at room air. The patient appeared comfortable with no signs of respiratory distress. These findings were consistent with those done in the emergency department; however, the CXR revealed an unanticipated finding of a foreign body inside the trachea approximately 2 cm above the carina, which appeared tubular (Figure 1). Further investigation of history revealed that the patient had an NPA placed in the last six months.

**Figure 2:**
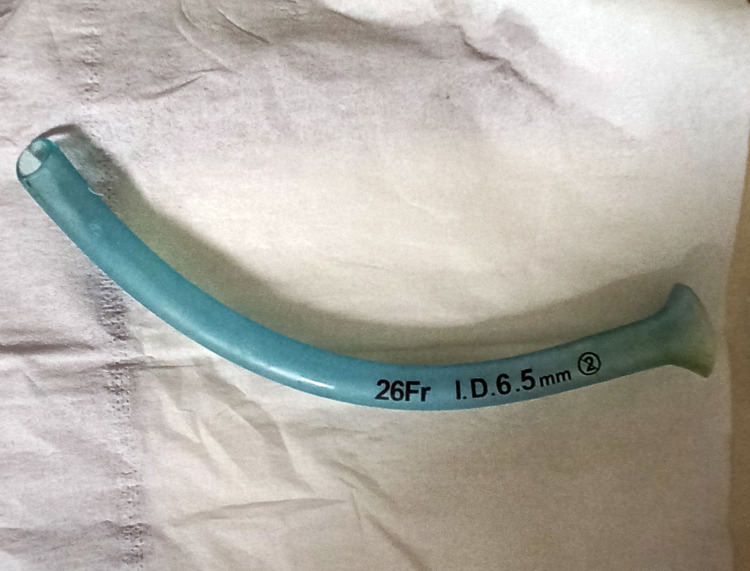
Retrieved NPA (6.5 mm ID) NPA, nasopharyngeal airway; ID, internal diameter

Careful direct laryngoscopy was done with a MAC 3 blade, which showed the tip of the NPA just below the epiglottis. Magill forceps were used to successfully retrieve the NPA, which had a 6.5 mm internal diameter (ID) (Figure 2).

**Figure 3:**
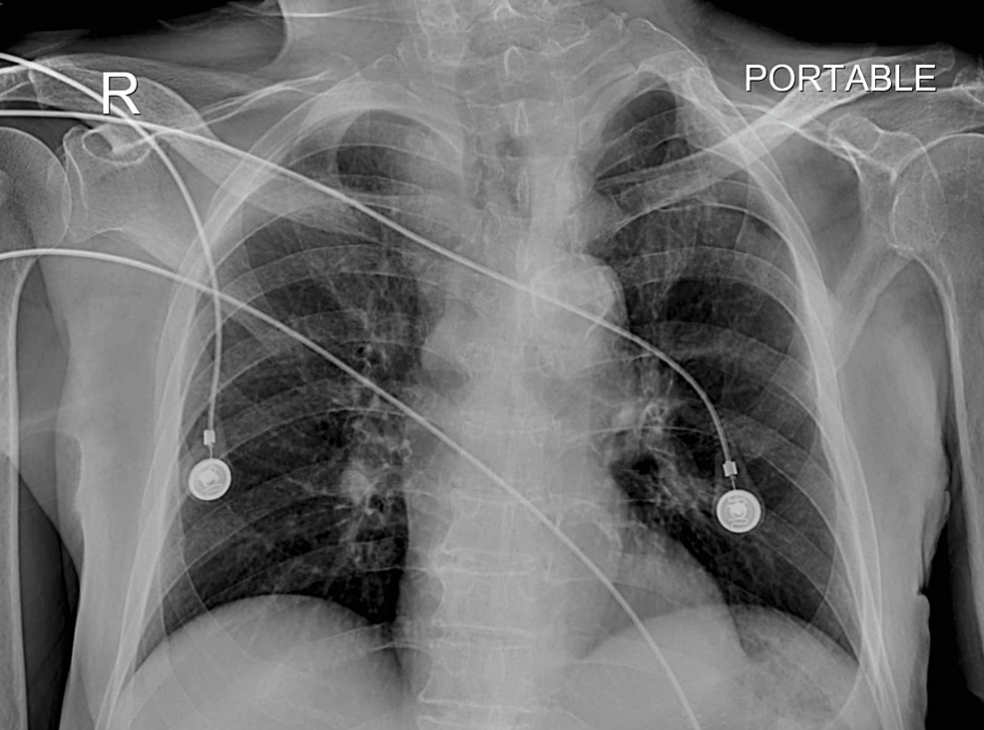
Follow-up CXR showing patent trachea CXR, chest X-ray

An adequately sized NPA of 7.5 ID was later inserted. A safety pin was inserted through the flange of the NPA and the family was counseled in detail regarding the care of NPA. Furthermore, the patient was kept under observation for the next 12 hours in the ICU, and a follow-up CXR was ordered (Figure 3). A patent trachea was documented, and there were no obvious findings suggestive of aspiration pneumonia, following which a decision was made to step down the patient from the ICU.

## Discussion

NPAs are routinely used as safe adjuncts for airway management. Despite their perceived safety, certain complications may pose a significant challenge. One such clinical scenario is where an NPA unknowingly may migrate into the trachea and consequently result in respiratory distress and hypoxia [1]. It is of paramount importance that prompt management is done via utilizing X-rays and flexible bronchoscopy to localize and remove the dislodged NPA [2].

We present a unique case report detailing an unusual complication where a NPA migrated into the trachea causing no respiratory distress or hypoxia. The NPA was discovered only upon reviewing the routine pre-admission CXR and in-depth clinical history. It is our assessment that an absent gag reflex and the spontaneous positioning at approximately 2 cm above the carina resulted in the NPA patently cannulating the trachea and, therefore, causing no clinically recognizable signs such as stridor or respiratory distress. NPA of 7.5 mm ID would have been a reasonable choice in this particular patient [3,4]. Barriers such as safety pins and adequate training of medical staff should be ensured in all patients being managed with NPAs, especially in patients with neurological impairment [5].

## Conclusions

This case emphasizes the importance of proper sizing, careful insertion, and vigilant monitoring of NPAs, especially in patients with altered mental status. We recommend the usage of barriers such as safety pins and training medical staff to prevent an inadvertent migration of NPAs into the trachea. These proactive measures are essential for patient safety and preventing potentially life-threatening complications associated with airway obstruction.
